# Supplementary material for: Socio-Economic Inequalities in Child Stunting Reduction in Sub-Saharan Africa
Source: Nutrients. 2020 Jan 18;12(1):253. doi: 10.3390/nu12010253 (PMC7019538; doi:10.3390/nu12010253)
Supplement: Supplementary file 1 [file nutrients-12-00253-s001.pdf]

Supplementary file S1: Estimates of minimum dietary diversity, minimum meal frequency, and minimum adequate diet for children 6-23 months of age

A. Children 6-23 months fed 5+ food groups

			Residence		Wealth quintile				
**Country**	**Survey**	**Total**	**Urban**	**Rural**	**Lowest**	**Second**	**Middle**	**Fourth**	**Highest**
Angola	2015-16 DHS	29.1	33.1	22.9	19.3	23.6	29.2	34.7	44.7
Benin	2017-18 DHS	25.2	23.5	26.3	23	25.2	27.1	25.2	25.5
Benin	2011-12 DHS	28.7	33.3	25.4	20.4	23	28	30.8	41.2
Burkina Faso	2010 DHS	5.1	11.3	3.7	3.5	3.2	3.9	4.1	12.7
Burundi	2016-17 DHS	17.6	41.2	15.4	8.7	10	17.1	20.2	38.1
Burundi	2010 DHS	16.5	29.2	15.3	11.6	12.3	11.2	21.8	27.3
Cameroon	2011 DHS	25	33	18.9	7.7	18.5	30.1	31.7	42.7
Chad	2014-15 DHS	9.3	18	7.4	4.6	5.2	6.8	12.7	20.5
Comoros	2012 DHS	21.5	28.6	18.7	18.9	20.1	21.8	24.5	23.1
Congo	2011-12 DHS	15.9	17.6	13.2	11.6	15	19.8	16	18.5
DRC	2013-14 DHS	17.2	25.2	13.6	10.5	14.5	13.1	22.9	28.1
Cote d'Ivoire	2011-12 DHS	7.6	12.9	4.3	2.4	5.5	6.6	10.9	16.6
Ethiopia	2016 DHS	12.5	28.1	10.3	6.5	10.1	11.8	12.6	25.6
Ethiopia	2011 DHS	4.4	10.1	3.5	1.3	4.4	2.7	3.7	11.6
Gabon	2012 DHS	17.8	18.5	14.4	11.9	18	19.9	18.7	22.1
Gambia	2013 DHS	10.3	14	7.3	7.1	7.8	9.6	14.7	14
Ghana	2014 DHS	24.4	28.9	20.7	15.5	14.8	24.5	29	42.2
Guinea	2018 DHS	13.8	20.5	10.9	7.4	10.1	11.1	19.4	24
Guinea	2012 DHS	6.4	13.4	3.7	2.8	2.3	4.6	8.3	16.3
Kenya	2014 DHS	36.3	50.4	28.3	18.2	25.8	33.7	50.7	57.7
Lesotho	2014 DHS	16.9	26.5	13.6	8.2	12	13	21.2	37
Liberia	2013 DHS	8.3	9	7.7	7.6	4.6	7.9	12.5	10.2
Malawi	2015-16 DHS	22.9	39	20.4	14.3	19.3	21.7	24.6	42
Mali	2018 DHS	21.8	38.3	17.3	17.3	13.7	14.9	24.4	41.8

Mali	2012-13 DHS	18.5	27.2	16.3	12.8	14.7	15.4	20.5	30.3
Mozambique	2011 DHS	27.7	26	28.3	30.5	26.9	31	21.7	27.4
Namibia	2013 DHS	24.9	39	12.8	9.2	14.5	23.8	37.4	52.7
Niger	2012 DHS	8.5	26.6	5.4	3.5	4.3	4.4	8	22
Nigeria	2018 DHS	22.6	29	18.6	16.3	16.3	19.7	27.5	37.3
Nigeria	2013 DHS	15.5	22.1	11.8	8	10.6	16.4	21	24.8
Rwanda	2014-15 DHS	28.2	44.5	24.9	14.9	21.5	24.1	41.2	47
Rwanda	2010 DHS	24.6	45.3	22.1	16.9	20	23.1	23.4	46.7
Senegal	2017 DHS	20.3	32.7	13.1	8.1	11.7	17.8	31.4	41.9
Senegal	2016 DHS	12.6	16.9	10.4	8.7	10.8	13.8	16	16.8
Senegal	2015 DHS	16	16.3	15.8	10.3	16.9	18.3	17.4	20.1
Senegal	2014 DHS	16.3	18.7	14.5	10.5	14.8	23.9	9.6	24.5
Senegal	2012-13 DHS	15.9	26.3	10.8	7.6	10.6	16	18.2	33.1
Senegal	2010-11 DHS	23.6	32.4	18	12.9	16	26.5	24.9	44.7
Sierra Leone	2013 DHS	13.4	20	11.2	8.9	11.4	11.2	15	25.1
South Africa	2016 DHS	39.9	44.2	33.5	37.7	33.1	39.7	43.1	51.4
Tanzania	2015-16 DHS	21.5	32.9	17.2	12.7	12.9	18.5	28.1	40.4
Togo	2013-14 DHS	18.3	24.6	14.8	17.1	14.2	12	19.3	30.1
Uganda	2016 DHS	24.9	28.9	23.8	18.8	23.6	22.8	26.5	33.9
Uganda	2011 DHS	10.2	18.4	8.9	4	6.1	11.1	12.9	19.7
Zambia	2013-14 DHS	18.4	24.4	15.3	13.7	15.6	15.3	20.7	32.2
Zimbabwe	2015 DHS	22.6	37.4	17	13.1	17.8	18.2	28.5	43.7
Zimbabwe	2010-11 DHS	17.9	28.4	13.7	10.1	12.2	18.3	20.9	33.2

Percentage of children age 6-23 months fed five or more food groups. The food groups are 1. breastmilk 2. infant formula, milk other than breast milk, cheese or yogurt or other milk products; 3. foods made from grains, roots, and tubers, including porridge and fortified baby food from grains; 4. vitamin A-rich fruits and vegetables (and red palm oil); 5. other fruits and vegetables; 6. eggs; 7. meat, poultry, fish, and shellfish (and organ meats); 8. legumes and nuts.

B. Children 6-23 months of age meeting the minimum meal frequency (MMF)

			Residence		Wealth quintile				
**Country**	**Survey**	**Total**	**Urban**	**Rural**	**Lowest**	**Second**	**Middle**	**Fourth**	**Highest**
Angola	2015-16 DHS	32.4	36.2	26.6	28.3	23.6	30.5	36	50.4
Benin	2017-18 DHS	44.9	45.6	44.5	42.3	43.2	46.9	43.3	49.3
Benin	2011-12 DHS	41.8	45.2	39.3	36.7	37.7	40.8	44.7	48.8
Burkina Faso	2010 DHS	36.8	38.3	36.5	36	36.4	33.5	37.6	42.1
Burundi	2016-17 DHS	39.2	59.2	37.4	34	32.1	40.3	36.7	58
Burundi	2010 DHS	31.9	40.1	31.1	28.1	29.4	28.5	34.2	40.4
Cameroon	2011 DHS	28.2	25.7	30.2	38.8	29.6	21.5	23.7	25.4
Chad	2014-15 DHS	37.1	38.7	36.8	32.3	31.1	39.5	45	39.4
Comoros	2012 DHS	28.7	28	29	21.3	28.1	37.4	24.1	32.5
Congo	2011-12 DHS	23.6	21.7	26.7	27.3	20.2	22.9	27.8	18.9
DRC	2013-14 DHS	34.6	30.7	36.4	35.3	37.5	34.6	33.6	31.2
Cote d'Ivoire	2011-12 DHS	40	41.8	38.9	35.7	45.7	39	35.9	46.3
Ethiopia	2016 DHS	45.1	59.2	43.1	38.3	41.4	48.7	45	55.2
Ethiopia	2011 DHS	48.4	52	47.8	41.9	47.1	51.3	50.8	53.2
Gabon	2012 DHS	29.9	30	29.3	29.5	26	34.9	27.5	31.9
Gambia	2013 DHS	57.5	58.4	56.8	54.5	56.5	58	63.5	55.5
Ghana	2014 DHS	43.3	44.5	42.2	41	35.8	47.3	46.1	47.9
Guinea	2018 DHS	23.7	30.2	20.9	18.3	22.2	20.4	24.6	35.3
Guinea	2012 DHS	30.1	37	27.5	25.2	24.4	25.3	38.9	39.7
Kenya	2014 DHS	50.6	58.9	45.9	41.8	45.8	51.2	53.6	62.8
Lesotho	2014 DHS	60.4	59.4	60.7	59.4	66.1	59.9	54.3	63.6
Liberia	2013 DHS	29.8	34.3	25.1	22.9	29.4	29.7	30.8	40.7
Malawi	2015-16 DHS	29.2	37.1	27.9	24.2	27.8	30.6	30.6	36.1
Mali	2018 DHS	30.1	36.9	28.2	25.7	28	31.7	28	37.5
Mali	2012-13 DHS	27.7	29.8	27.1	28.2	23.4	26.7	28.2	32.5
Mozambique	2011 DHS	41.1	46.1	39.3	43.1	35.5	38.1	40.6	51.6

Namibia	2013 DHS	40	48.9	32.5	25.4	40.3	39.1	40.2	64.1
Niger	2012 DHS	51.1	49.9	51.4	44.8	50.3	51.7	56.5	51.3
Nigeria	2018 DHS	41.8	41.5	42.1	42.6	41.6	39.5	40.1	46
Nigeria	2013 DHS	57.9	56.6	58.5	60.2	60.1	57.3	56.3	54.3
Rwanda	2014-15 DHS	47.1	52.5	46.1	37.5	45.4	44.6	55.3	57.7
Rwanda	2010 DHS	50.7	56.9	50	45.7	47.3	46.5	56.5	61.7
Senegal	2017 DHS	30.6	31.4	30	27.5	29.8	31	28.8	37.4
Senegal	2016 DHS	34.7	40.3	31.9	31.9	29.8	29.4	38.6	48.9
Senegal	2015 DHS	40.5	44	38.9	36.6	39.4	40.1	42.1	47.7
Senegal	2014 DHS	34.6	36.1	33.5	32.1	34.1	35.3	29.1	44
Senegal	2012-13 DHS	35	43.2	30.9	32	33.7	33.7	32.7	45
Senegal	2010-11 DHS	34	34	33.9	35.1	35.4	34.3	30.5	34.4
Sierra Leone	2013 DHS	38.7	46.2	36.2	31.1	36.5	37.6	43.5	50.2
South Africa	2016 DHS	50.2	52.9	46	41.5	49.4	45.3	56.6	65.6
Tanzania	2015-16 DHS	39.9	36.4	41.2	42.1	39.6	42.1	35.7	39.3
Togo	2013-14 DHS	45.9	42.5	47.8	56.9	45.2	42.5	38	47.3
Uganda	2016 DHS	41.4	43.3	40.8	35.8	41	41.7	40.8	48.2
Uganda	2011 DHS	44.7	48.9	44.1	35	43.3	48.8	45	54.7
Zambia	2013-14 DHS	42.2	46.3	40	35	40.6	42	47.2	51.2
Zimbabwe	2015 DHS	35.8	41.2	33.7	28.1	33.1	37	38.1	48.6
Zimbabwe	2010-11 DHS	45.2	52.5	42.2	42.3	40.4	44.2	44.7	58

Minimum Meal Frequency (MMF): Percentage of children age 6-23 months fed the minimum meal frequency defined as receiving solid or semi-solid food at least twice a day for infants 6-8 months and at least three times a day for children 9-23 months

C. Children 6-23 months of age meeting the minimum adequate diet (MAD)

			Residence		Wealth quintile				
Country	Survey	Total	Urban	Rural	Lowest	Second	Middle	Fourth	Highest
Angola	2015-16 DHS	13.1	15.5	9.4	8.2	8.9	14.1	14.9	22.9
Benin	2017-18 DHS	14.3	14.1	14.5	14.9	14.4	14	13.2	15.2
Benin	2011-12 DHS	15.8	18.6	13.8	12.1	11.5	14.5	16.8	23.7
Burkina Faso	2010 DHS	3.1	6.4	2.4	2.5	1.8	2.1	2.6	7.6
Burundi	2016-17 DHS	9.8	28.1	8.1	3.6	4.4	10.4	10.5	24.7
Burundi	2010 DHS	8.8	18.3	7.8	5.1	5.5	6.2	11.7	16.4
Cameroon	2011 DHS	5.5	6.9	4.5	2.9	5	5.9	7.2	7.4
Chad	2014-15 DHS	5.7	8.5	5	3.2	2.5	4.4	9.9	10
Comoros	2012 DHS	5.7	7.7	4.9	4.3	4.9	6.3	6.1	7.5
Congo	2011-12 DHS	4.9	4.8	5.1	4.6	4.4	7.6	4.4	3.5
DRC	2013-14 DHS	8.4	12	6.8	5.2	8.1	6	10	13.9
Cote d'Ivoire	2011-12 DHS	4.6	6.9	3.1	2.2	4.5	3	6.4	8.4
Ethiopia	2016 DHS	7.3	18.5	5.7	2.8	6.7	8	5.2	16.2
Ethiopia	2011 DHS	4.1	9.2	3.2	1.2	3.8	2.7	3.6	10.9
Gabon	2012 DHS	4.7	4.6	5.2	6.1	3.9	3.2	3.7	7.5
Gambia	2013 DHS	8.3	11.1	6	5.4	7.1	8.1	12.4	9.4
Ghana	2014 DHS	13.3	14.4	12.3	11.4	8	15.7	11.4	21.7
Guinea	2018 DHS	4.3	7.3	3	1.1	2.8	4.3	5.7	8.7
Guinea	2012 DHS	3.7	6.9	2.5	1.7	0.8	3	5.8	8.3
Kenya	2014 DHS	21.8	31.2	16.5	10.3	15.9	19.3	29.7	36.5
Lesotho	2014 DHS	11.3	18.3	9	5.4	8.4	6.4	16.6	24.6
Liberia	2013 DHS	4.1	5.3	2.9	3	1.7	3.3	7.5	6.5
Malawi	2015-16 DHS	8.1	16	6.8	3.6	6.4	7.2	10.6	16.5
Mali	2018 DHS	8.9	18.4	6.4	6	5.4	6	9.8	19
Mali	2012-13 DHS	7.7	11.1	6.8	7	4.2	5.7	8.5	13.6
Mozambique	2011 DHS	13	12.3	13.3	16.9	12.1	13.8	7.7	13.7

Namibia	2013 DHS	12.4	20.3	5.7	2.7	6.6	11	18.2	32
Niger	2012 DHS	5.5	15.3	3.9	2.7	3.1	3.5	5.5	12.7
Nigeria	2018 DHS	10.6	14	8.5	7.5	8	8.1	12.6	18.8
Nigeria	2013 DHS	10.2	13.7	8.2	6	7.8	10.4	13.7	14.9
Rwanda	2014-15 DHS	18.1	29.8	15.7	7.6	13.7	15.7	28.1	31
Rwanda	2010 DHS	16.8	32.1	15	11.3	12.2	14	17.5	34.8
Senegal	2017 DHS	8	10.9	6.3	4.4	5	8.3	11.3	13.5
Senegal	2016 DHS	6.6	9.5	5.2	4	5.5	5.7	9.1	11.4
Senegal	2015 DHS	10.2	10.9	9.9	6.8	10.8	9.8	11.5	14.1
Senegal	2014 DHS	8	8.6	7.6	5.7	7.8	11.3	2.7	13.2
Senegal	2012-13 DHS	9.6	15.6	6.6	5.4	8.3	7.9	10.3	19.3
Senegal	2010-11 DHS	10.1	13.5	8	6	8.1	11.3	11.2	16.3
Sierra Leone	2013 DHS	6.9	11.6	5.4	2.9	5.6	5.9	7.9	15.8
South Africa	2016 DHS	22.5	25.2	18.5	17.3	16	22.5	25.9	38.5
Tanzania	2015-16 DHS	8.7	12	7.4	6.4	5.8	7.1	9.4	16.4
Togo	2013-14 DHS	12.1	13.7	11.2	13.7	11.4	8.7	10.7	16.5
Uganda	2016 DHS	14.2	16.2	13.7	10.3	13.9	12.9	14.4	20.1
Uganda	2011 DHS	5.9	10.1	5.3	2.7	4.1	7.8	4.9	11.5
Zambia	2013-14 DHS	10.5	13.3	9	7.3	9.7	9.6	12.1	16.5
Zimbabwe	2015 DHS	8.1	13.5	6	2.5	5.5	8.2	10.2	18.5
Zimbabwe	2010-11 DHS	10.9	17.7	8.2	6.4	6.5	11.2	14.2	19.1
